# Phosphorus addition changes belowground biomass and C:N:P stoichiometry of two desert steppe plants under simulated N deposition

**DOI:** 10.1038/s41598-018-21565-w

**Published:** 2018-02-21

**Authors:** Juying Huang, Hailong Yu, Jili Liu, Chengke Luo, Zhaojun Sun, Kaibo Ma, Yangmei Kang, Yaxian Du

**Affiliations:** 1 Institute of Environmental Engineering, Ningxia University, Yinchuan, 750021 China; 2 Ningxia (China-Arab) Key Laboratory of Resource Assessment and Environment Regulation in Arid Region, Yinchuan, 750021 China; 3 College of Resources and Environment, Ningxia University, Yinchuan, 750021 China

## Abstract

Many studies have reported that increasing atmospheric nitrogen (N) deposition broadens N:phosphorus (P) in both soils and plant leaves and potentially intensifies P limitation for plants. However, few studies have tested whether P addition alleviates N-induced P limitation for plant belowground growth. It is also less known how changed N:P in soils and leaves affect plant belowground stoichiometry, which is significant for maintaining key belowground ecological processes. We conducted a multi-level N:P supply experiment (varied P levels combined with constant N amount) for *Glycyrrhiza uralensis* (a N fixing species) and *Pennisetum centrasiaticum* (a grass) from a desert steppe in Northwest China during 2011–2013. Results showed that increasing P addition increased the belowground biomass and P concentrations of both species, resulting in the decreases in belowground carbon (C):P and N:P. These results indicate that P inputs alleviated N-induced P limitation and hence stimulated belowground growth. Belowground C:N:P stoichiometry of both species, especially *P. centrasiaticum*, tightly linked to soil and green leaf C:N:P stoichiometry. Thus, the decoupling of C:N:P ratios in both soils and leaves under a changing climate could directly alter plant belowground stoichiometry, which will in turn have important feedbacks to primary productivity and C sequestration.

## Introduction

It is well known that anthropogenic activities, such as fossil fuel combustion, fertilizer use and intensive animal husbandry have produced a large number of nitrogen-containing compounds, which have resulted in increasing atmospheric nitrogen (N) deposition^1^. It has been estimated that the global production of reactive N increased from 1.50 × 10^13^ g N year^−1^ in 1860 to 1.65 × 10^14^ g N year^−1^ in 2000^2^. In China, N deposition amounts have increased at the average rate of 0.041 g m^−2^ year^−1^ during 1980–2010^3^ and are expected to continue over the next several decades^4^. The ongoing increase in N deposition has accelerated N cycling in terrestrial ecosystems and also altered phosphorus (P) availability to plants^5–7^. Phosphorous has been demonstrated to limit plant growth in most terrestrial ecosystems, either individually or in combination with N^8^. Thus, the increasing P limitation would affect primary production and P cycling in P-limited ecosystems. This raises two fundamental questions under N deposition: (1) how P addition affects P uptake and consequently C:N:P stoichiometry in plants and (2) whether or not P addition alleviates N-induced P limitation for plant growth.

Plant belowground and aboveground processes in plant-soil systems are tightly interlinked and their associations greatly influence ecosystem functions^9,10^. At an individual level, plant belowground organs uptake nutrients from soils and hence link plants and soils together^11^. Changes of soil nutrient availability will directly affect nutrient absorption and assimilation in plant belowground organs, thereby influencing C, N and P relationships in plant leaves^12^. Therefore, to better understand the effects of P addition on plant C:N:P stoichiometry under N deposition, it is necessary to consider the changes in soil properties and leaf eco-physiological characteristics while also taking into account the nutrient processes in plant belowground organs^11^. To date, much of the attention regarding nutrient dynamics and their relationships have been focused on soils and plant leaves. However, a clear explanation on the responses of C:N:P stoichiometry in plant belowground organs to P addition under N deposition is still lacking. If plant belowground C:N:P stoichiometry do change greatly, then plant belowground growth will be altered accordingly. Unfortunately, how plant belowground stoichiometry regulates belowground biomass accumulation under P addition combined with N has not been fully explored yet.

C:N:P ecological stoichiometry is a new approach for studying the interaction between plants and soils under changing global climate^13,14^. It is commonly suggested that elemental stoichiometry is inherently steady, which is important for maintaining ecosystem stability. However, the balance of elemental stoichiometry in soils and plant leaves tend to be decoupled by intensifying climate change^15–17^. In some N limited ecosystems, short-term N addition had inconsistent influence on C status but increased soil N and P availability, consequently, promoting leaf N uptake and synergistic absorption of P^18^. With gradual input of N, N:P becomes disproportionate and plant demand for P increases^19^. These nonsynchronous changes of the three elements generally result in increasing N:P but decreasing C:N in soils and leaves^20,21^. The cycles of C, N and P in plant-soil systems are reciprocal interchanged among aboveground and belowground organs of plants and soils^22^. Thus, the decoupling of C:N:P relationships in soils and leaves are supposed to affect the stoichiometric balance in plant belowground organs, which closely associates with key belowground ecological processes^11^. However, this conjecture has not been widely tested in terrestrial ecosystems, especially in desert steppe ecosystems.

The Ningxia Hui Autonomous Region, China, covers a large area of desert steppe ecosystem, which is characterized by low soil N availability and critical load of N deposition^23^. A recent study estimates that N deposition has exceeded 4.0 g m^−2^ year^−1^ in some places of this region during 2000–2010^24^. Therefore, the growth and stoichiometry of plants growing there should be affected accordingly. The observations from our previous field experiment, which was conducted in 2010 in a desert steppe of Ningxia, showed that both plant growth and species number decreased while N addition exceeded 10 g m^−2^ yr^−1 ^^25,26^. We speculated that over 10 g N m^−2^ yr^−1^ might widen N:P and accelerate P limitation for most species. Thus, we designed the present experiment which involved six N:P treatments during 2011–2013. After 3-year of treatments, we analyzed plant belowground biomass and stoichiometry and their relationships with C:N:P ratios in both soils and green leaves, respectively. Our objectives were to explore, (1) how P addition affects plant belowground stoichiometry under simulated N deposition, (2) if and to what extent P addition alleviates N-induced P limitation for plant belowground growth and (3) how the decoupling of C:N:P ratios in both soils and leaves affect plant belowground stoichiometry. Our results will be helpful to identify the mechanism controlling elemental dynamics within soils and plants in desert steppe ecosystems.

## Results

### Changes in belowground biomass and root/rhizome to shoot ratio (RSR)

**Figure 1:**
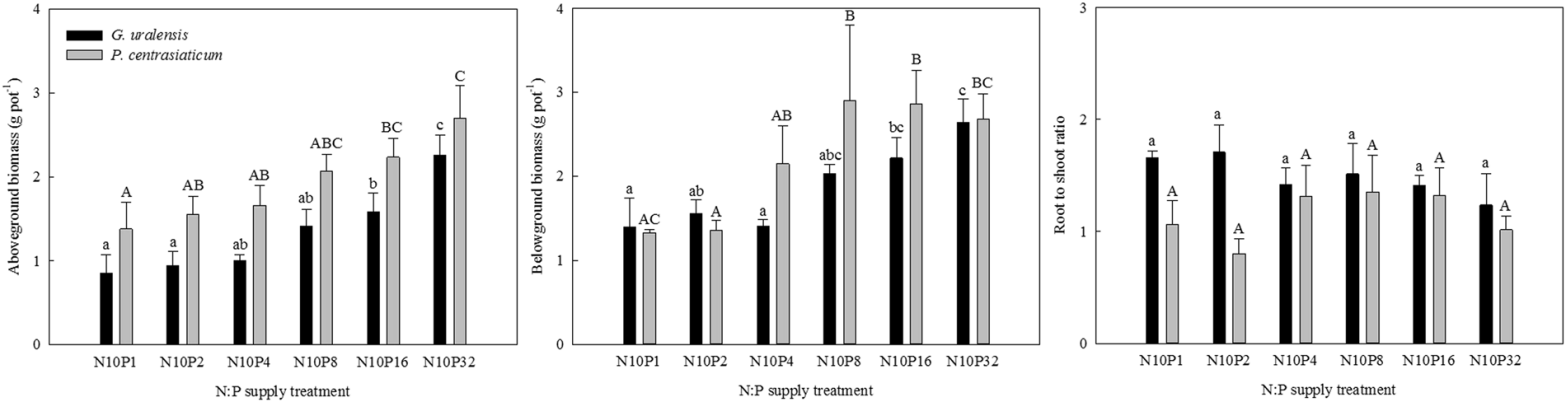
Effects of N:P supply treatments on belowground biomass and root/rhizome to shoot ratio of the two species. Lowercases above black bars and uppercases above grey bars represent significant differences (*P* < 0.05) among the N:P treatments for *G. uralensis* and for *P. centrasiaticum*, respectively.

Both N:P supply and species had significant effects on belowground biomass, whereas only species had a significant effect on RSR (Table 1, *P* < 0.01). Specifically, low P addition (high N:P supply) had insignificant effects on belowground biomass of the two species, while high P addition (≥16 g m^−2^ year^−1^ for *G. uralensis* and ≥8 g m^−2^ year^−1^ for *P. centrasiaticum*, respectively) greatly increased belowground biomass of both species (Fig. 1). In contrast, there were no significant differences in RSR among six N:P supply treatments. On average, *G. uralensis* had relatively low belowground biomass but high RSR compared with *P. centrasiaticum* (Fig. 1 and Table 2).

**Table 1:** Effects of N:P supply treatment and species and their interaction on belowground growth and C:N:P stoichiometry of the two species (Two-Way ANOVA).

Source		d.f.	*F*	*P*
Belowground biomass	N:P supply	5	7.657	0.000
	Species	1	15.164	0.000
	N:P supply × Species	5	0.153	0.977
Root/Rhizome to shoot ratio	N:P supply	5	0.739	0.602
	Species	1	7.974	0.009
	N:P supply × Species	5	0.736	0.604
Belowground C concentration	N:P supply	5	1.037	0.419
	Species	1	54.333	0.000
	N:P supply × Species	5	0.758	0.589
Belowground N concentration	N:P supply	5	5.995	0.000
	Species	1	38.218	0.000
	N:P supply × Species	5	0.578	0.716
Belowground P concentration	N:P supply	5	66.221	0.001
	Species	1	12.539	0.002
	N:P supply × Species	5	24.242	0.001
Belowground C:N ratio	N:P supply	5	5.433	0.002
	Species	1	60.913	0.000
	N:P supply × Species	5	1.629	0.191
Belowground C:P ratio	N:P supply	5	96.161	0.000
	Species	1	55.963	0.000
	N:P supply × Species	5	20.602	0.000
Belowground N:P ratio	N:P supply	5	40.200	0.000
	Species	1	17.994	0.000
	N:P supply × Species	5	3.900	0.000

**Table 2:** *F* values of the differences between the two species under each N:P supply treatment (Independent-Samples T test).

Indices	N10P1	N10P2	N10P4	N10P8	N10P16	N10P32
Belowground biomass	3.161	0.026	10.827**	5.338*	0.470	0.003
Root/rhizome to shoot ratio	3.293	0.021	0.312	1.431	0.117	1.594
Belowground C concentration	3.106	0.431	3.072	0.000	6.823*	0.520
Belowground N concentration	6.258*	2.324	2.183	1.547	0.020	2.968
Belowground P concentration	0.522	0.000	3.798	0.021	2.218	10.077**
Belowground C:N ratio	0.705	1.661	1.641	0.001	2.758	10.178
Belowground C:P ratio	3.707	3.018	4.062	1.182	0.065	5.991*
Belowground N:P ratio	2.138	5.623*	3.821	3.319	0.001	0.486

*and ** represent effects are significant at the 0.01 and 0.05 levels, respectively.

### Changes in C:N:P stoichiometry of belowground organs

**Figure 2:**
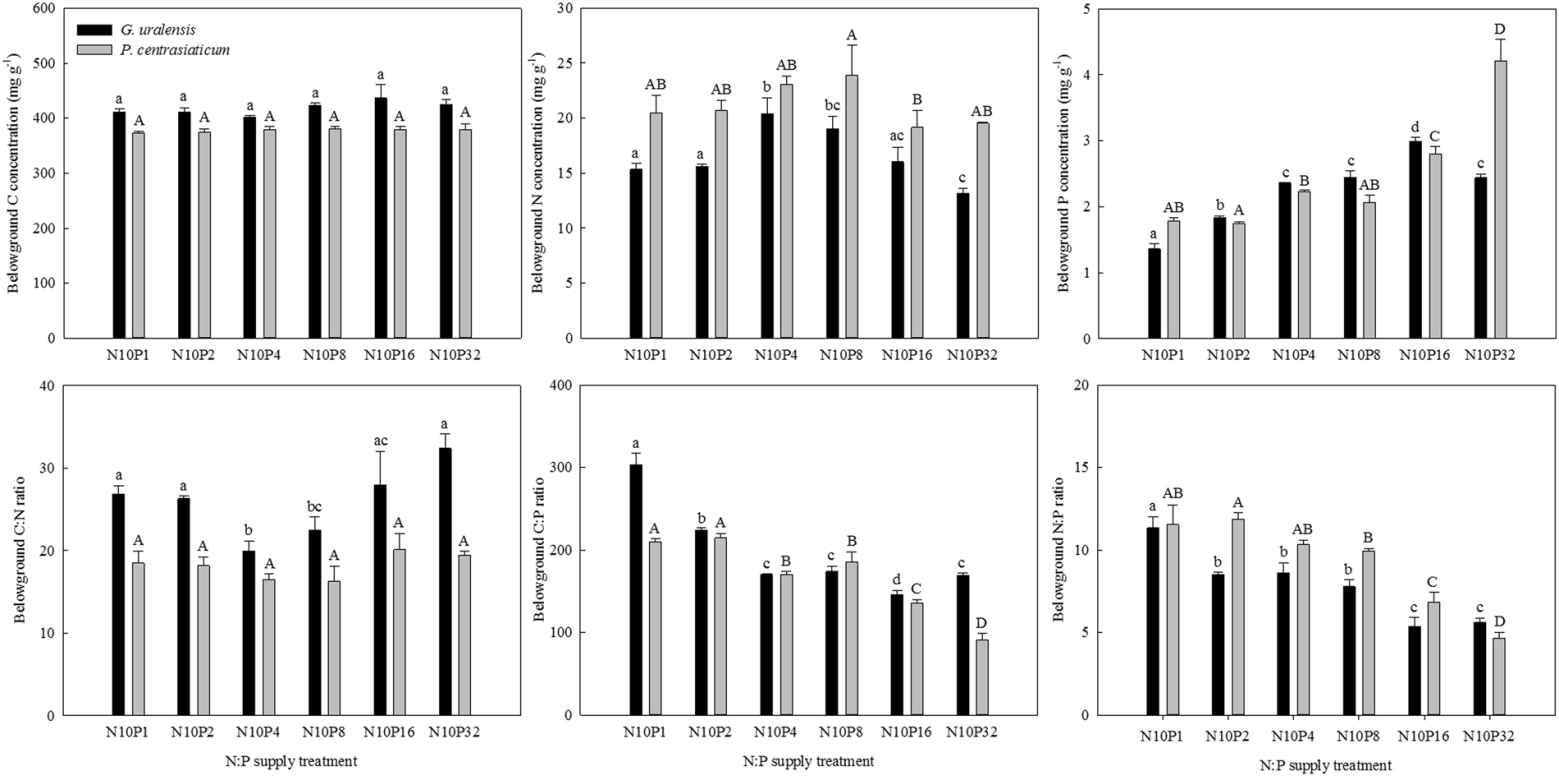
Effects of N:P supply treatments on belowground C:N:P stoichiometry of the two species. Lowercases above black bars and uppercases above grey bars represent significant differences (*P* < 0.05) among the N:P treatments for *G. uralensis* and for *P. centrasiaticum*, respectively.

Both N:P supply and species had large effects on belowground C:N:P stoichiometry (except belowground C), whereas their interaction only had significant effects on belowground P, C:P and N:P (Table 1, *P* < 0.01). Across the six N:P treatments, there was lack of significant trends in belowground C, N and C:N. In contrast, the belowground P of both species substantially increased with increasing P amount, consequently resulting in decreases of belowground C:P and N:P (Fig. 2). On average, *G. uralensis* had higher belowground C, C:N and C:P but lower N, P and N:P than *P. centrasiaticum* (Fig. 2 and Table 2).

### Changes in C:N:P stoichiometry in soils and green leaves

**Figure 3:**
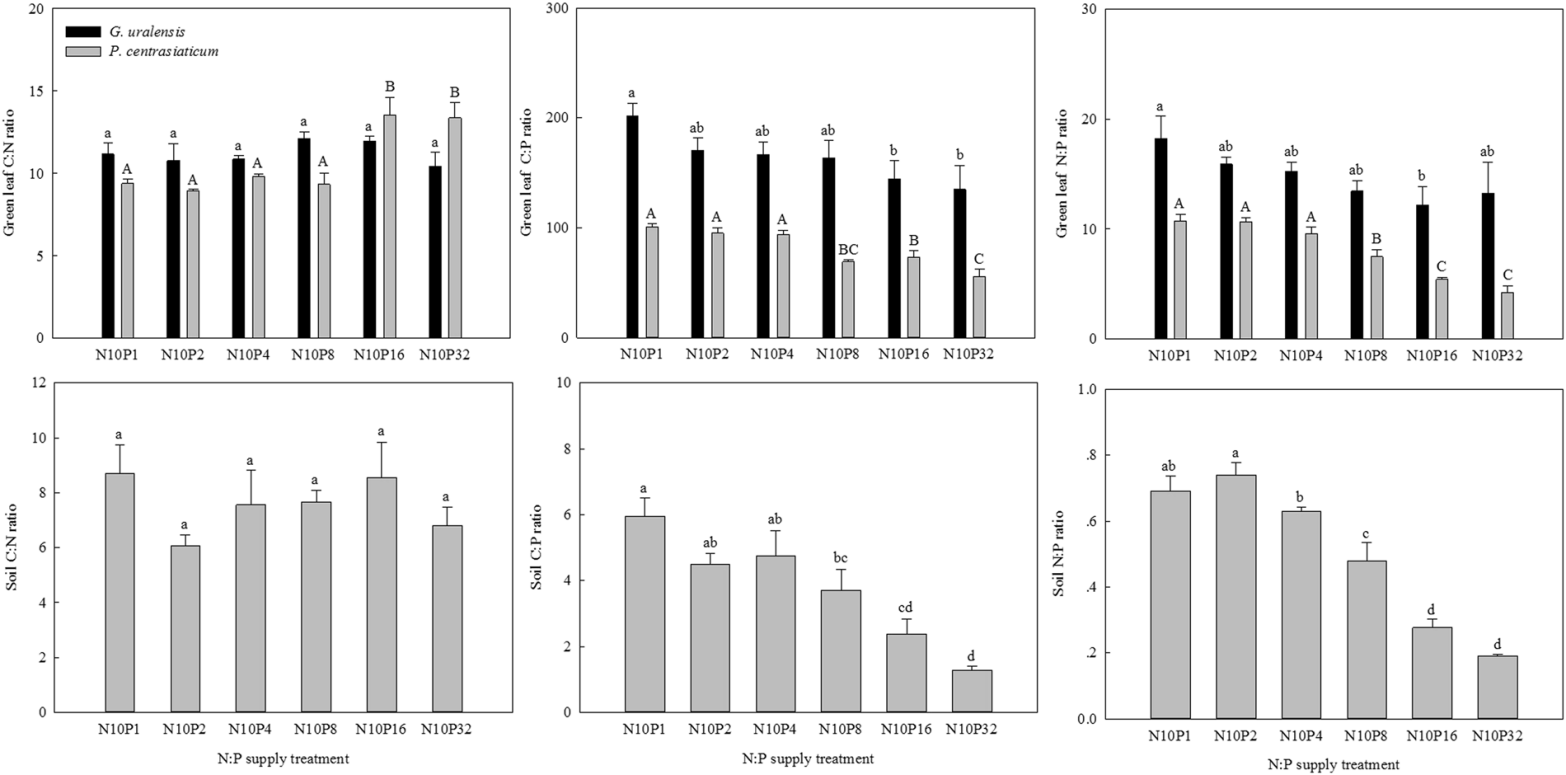
Effects of N:P supply treatments on C:N:P stoichiometry in both soils and green leaves of the two species. Lowercases above black bars and uppercases above grey bars represent significant differences (*P* < 0.05) among the N:P treatments for *G. uralensis* and for *P. centrasiaticum*, respectively.

Across the six N:P treatments, the C:P and N:P in soils and green leaves of both species generally decreased with increasing P amount. However, there was no clear effects on C:N both in soils and in green leaf of *G. uralensis* (Fig. 3). In general, the averages of C:N, C:P and N:P in green leaves of *G. uralensis* were higher than those in *P. centrasiaticum*.

### Relationships between plant belowground biomass and C:N:P stoichiometry

**Figure 4:**
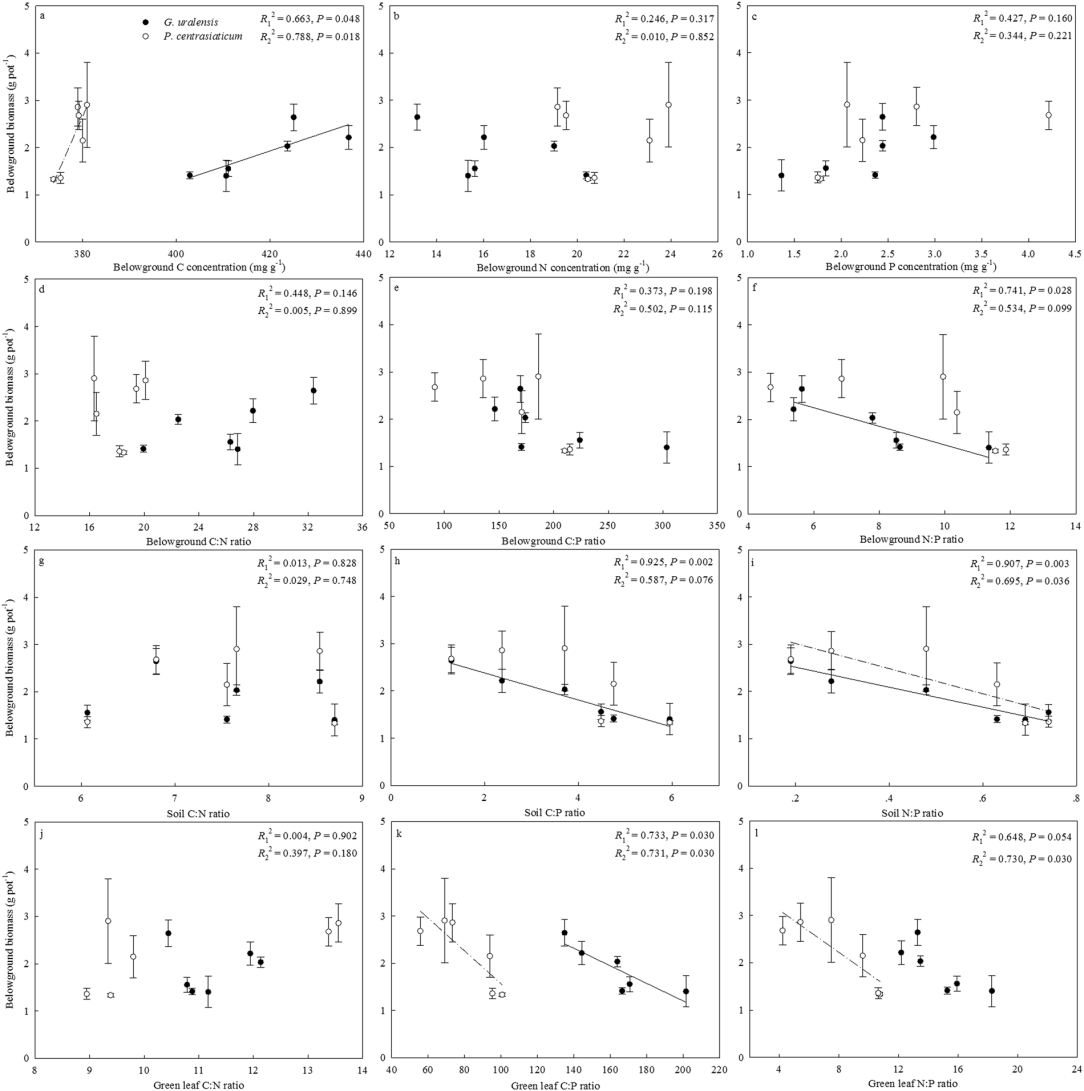
Relationships between belowground biomass and C:N:P stoichiometry. Black circles fitted with solid lines are for *G. uralensis* (*R*_1_^2^), white circles fitted with dot lines are for *P. centrasiaticum* (*R*_2_^2^), respectively. (**a**–**f**), (**g**–**i**) and (**j**–**l**) represent for the relationships between belowground biomass and belowground C:N:P, between belowground biomass and soil C:N:P and between belowground biomass and green leaf C:N:P, respectively.

Belowground biomass of *G. uralensis* was highly related to belowground C, belowground N:P, soil C:P, soil N:P and also green leaf C:P, respectively. For *P. centrasiaticum*, belowground biomass was only significantly related to belowground C, soil N:P, green leaf C:P and also green leaf N:P, respectively (Fig. 4).

### Relationships between plant belowground C:N:P stoichiometry and soil C:N:P stoichiometry

**Figure 5:**
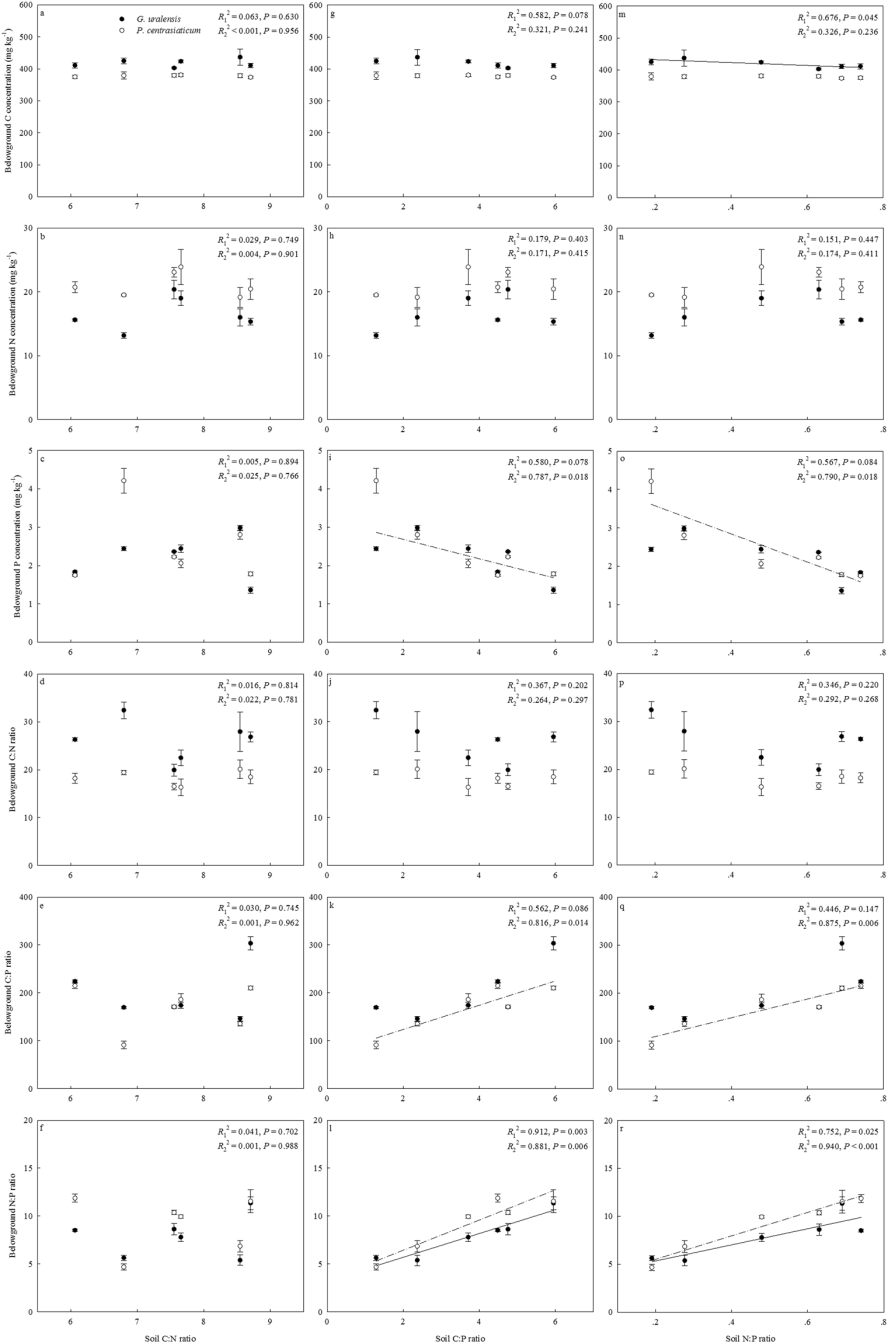
Relationships between belowground C:N:P stoichiometry and soil C:N:P stoichiometry. Black circles fitted with solid lines are for *G. uralensis* (*R*_1_^2^), white circles fitted with dot lines are for *P. centrasiaticum* (*R*_2_^2^), respectively. (**a**–**f**), (**g**–**l**) and (**m**–**r**) represent for the relationships between belowground C:N:P and soil C:N, between belowground C:N:P and soil C:P and between belowground C:N:P and soil N:P, respectively.

Positive linear relationships were found between belowground N:P and soil C:P and also between belowground N:P and soil N:P for *G. uralensis*, respectively (Fig. 5). Strong positive relationships were present between belowground C:P and soil C:P, between belowground C:P and soil N:P, between belowground N:P and soil C:P, as well as between belowground N:P and soil N:P for *P. centrasiaticum*, respectively.

In contrast, a negative linear relationship was present between belowground C and soil N:P for *G. uralensis*. The strong negative linear relationships were present between belowground P and soil C:P and also between belowground P and soil N:P for *P. centrasiaticum*, respectively.

### Relationships between plant belowground C:N:P stoichiometry and green leaf C:N:P stoichiometry

**Figure 6:**
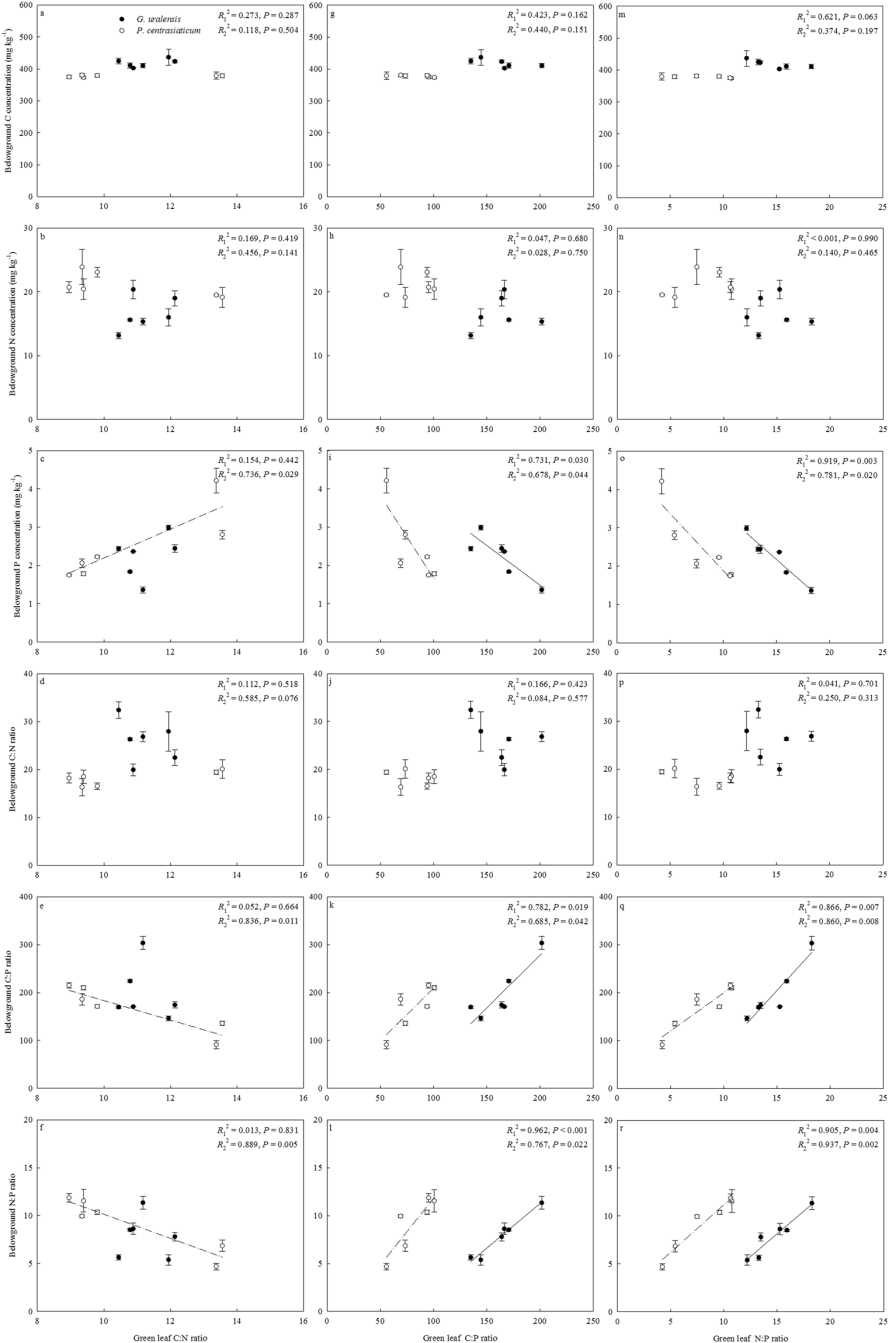
Relationships between belowground C:N:P stoichiometry and green leaf C:N:P stoichiometry. Black circles fitted with solid lines are for *G. uralensis* (*R*_1_^2^), white circles fitted with dot lines are for *P. centrasiaticum* (*R*_2_^2^), respectively. (**a**–**f**), (**g**–**l**) and (**m**–**r**) represent for the relationships between belowground C:N:P and green leaf C:N, between belowground C:N:P and green leaf C:P and between belowground C:N:P and green leaf N:P, respectively.

Positive linear relationships were found between belowground C:P and green leaf C:P, between belowground C:P and green leaf N:P, between belowground N:P and green leaf C:P and also between belowground N:P and green leaf N:P for *G. uralensis*, respectively (Fig. 6). Again, positive linear relationships were observed between belowground P and green leaf C:N, between belowground C:P and green leaf C:P, between belowground C:P and green leaf N:P, between belowground N:P and green leaf C:P, as well as between belowground N:P and green leaf N:P for *P. centrasiaticum*, respectively.

Negative linear relationships were found between belowground P and green leaf C:P and also between belowground P and green leaf N:P for *G. uralensis*. Negative relationships were also observed between belowground P and green leaf C:P, between belowground P and green leaf N:P, between belowground C:P and green leaf C:N, as well as between belowground N:P and green leaf C:N for *P. centrasiaticum*, respectively.

## Discussion

Previous research has suggested that an wider N:P supply (i.e. increasing N addition) can promote the growth of plants limited by N, whereas a reduced N:P supply (i.e. increasing P addition) is expected to increase the growth of plants limited by P^27^. We observed that low P addition (high experimental N:P supply) had little effects on the belowground biomass of both species. With the increase of P amounts (the thresholds were 16 g m^−2^ year^−1^ for *G. uralensis* and 8 g m^−2^ year^−1^ for *P. centrasiaticum*, respectively), the belowground biomass of both species gradually increased as well (Fig. 1), which is similar to the observations made from a grass species *Setaria sphacelata*^10^. One possible reason would be that the tested soil is poor in P availability, which was exacerbated by N addition. More importantly, the sampled soil is slightly alkaline, in which P is usually bound to carbonates^28^. Thus, low P addition might not be enough to overcome the carbonate barrier, rendering P unavailable to plants^29^. With increasing P addition, soil P availability increased and N-induced P pressures on plants were alleviated^5^, resulting in the increases of belowground growth. Our results indicate that moderate P addition is of great benefit to alleviate N-induced P limitation for the belowground growth of desert steppe plants. However, high P addition (exceeding 8 g P m^−2^ year^−1^) would cause P toxicity and N limitation^30^, which possibly slowed down the rate of belowground biomass accumulation of *P. centrasiaticum* (Fig. 1).

Plant biomass allocations between aboveground and belowground organs provide key information for connecting aboveground productivity and belowground C sequestration^31^. It reflects the balance between aboveground resources (light and CO_2_) and belowground resources (water and nutrients)^32^ and is highly plastic in response to environmental changes^33,34^. In nutrient poor ecosystems, plant growth will shift from nutrient limitation to light limitation under increasing nutrient enrichment^35^. To grow faster, plants often allocate more biomass to aboveground organs for achieving high leaf photosynthesis and thus producing more dry matter^27^. Some studies found that N addition mainly facilitated aboveground growth^36^ while P addition might be more beneficial for the biomass accumulation in belowground organs^37^. In the present study, the aboveground and belowground biomass of both species showed similar responses to increasing P addition under simulated N deposition as observed in other study^5^, thereby resulting in the insignificant differences of RSR among the treatments (Fig. 1). Compared with the previous studies involved with the single application of N or P^36,37^, our results may suggest that plant biomass allocation becomes more complicated under the combined application of N and P^34^.

Numerous studies have concluded that C:N:P ratios both in soils and in plant leaves would decouple under increasing nutrient enrichment^5,16,17,20^. Due to the differences in nutrient storage and metabolism function, plant belowground organs are considered to be less sensitive to environmental changes than plant leaves. Therefore, belowground C:N:P stoichiometry of both species were assumed to be less affected by P addition in the present study. However, we observed clear changes of C:P and N:P either in soils and green leaves or in belowground organs of both species across the treatments, which are inconsistent with the insignificant response of a grass species *S. sphacelata*^10^. One possible explanation would be that increasing P supply improved soil P availability and thus promoted the P uptake of both species as reported by another study^38^. This more positive response of belowground P than C and N consequently caused changes in belowground stoichiometric relationships. The increased belowground P uptake also reflected the alleviated P limitation for both species. Our results may support the findings that stoichiometric balance in belowground organs can also be destroyed by intensified environmental changes^36^. The altered belowground C:N:P relationships would in turn have important feedbacks to plant productivity and C sequestration through its influences on nutrient absorption, root turnover, microbial activity and other belowground ecological processes^36^.

Grasses are generally thought to possess lower N and P concentrations and be more easily affected by N and P fertilization than N-fixing species^8,39^. In the present study, we found that *P. centrasiaticum* had unexpectedly high belowground N (21.14 mg g^−1^ vs. 16.59 mg g^−1^) and P (2.47 mg g^−1^ vs. 2.24 mg g^−1^) concentrations compared with *G. uralensis*. On the one hand, the 10 g N m^−2^ yr^−1^ might have more positive effect on belowground N uptake of *P. centrasiaticum*, which is similar with the observations from previous study^39^. Accordingly, the belowground organs of *P. centrasiaticum* needed to absorb more P to balance the C:N:P relationship as reported in previous studies^18,40^. In this case, increased N availability would simultaneously stimulate the activity of P-mineralizing enzymes both in soils and on plant belowground organs^41^ and also increase the root/rhizome colonization of P-acquiring arbuscular mycorrhizal fungi^42^. Both of those approaches could provide more available P for belowground uptake and assimilation of *P. centrasiaticum*. On the other hand, the belowground growth of *P. centrasiaticum* was more greatly stimulated by increase of P addition, which promoted it uptake more N and P from soils. In summary, the greater positive response of N uptake to N and P fertilization likely resulted in higher N:P in belowground organs of *P. centrasiaticum* than that of *G. uralensis* (Fig. 2 and Table 2).

Previous studies have reported that C:N:P stoichiometry in soils and plants are tightly linked^9,11^. Plant C:N:P stoichiometry reflects litter decomposition quality and thus directly determines soil N and P availabilities, while soil C:N:P stoichiometry regulates microbial activity and also plant N and P uptakes. In the present study, we found C:P and N:P in belowground organs of the two species were highly related to C:P and N:P both in soils and in green leaves as reported in other studies^9,12,43^, especially *P. centrasiaticum*. The roots of N-fixing species can build symbiotic relationships with nodule bacteria, which in turn aid N-fixing species obtain N more easily and therefore depend less on soil N than other growth forms. Thus, the self-adjusting N strategy of *G. uralensis* probably contributed to less susceptibility of belowground C:N:P stoichiometry to changing C:N:P relationships in soils when compared with *P. centrasiaticum*. Our results suggest that the decoupling of C:N:P stoichiometry in soils and leaves under global climate change^15,17^ will directly affect the elemental stoichiometry balance in plant belowground organs, which will in turn have important influences on leaf nutrient uptake, soil nutrient availability and also nutrient cycling in plant-soil systems.

The C:N:P stoichiometry in both soils and plants could indicate nutrient limitation patterns and also relate to nutrient use efficiency^44,45^. Therefore, the changes of C:N:P relationships in soils and plants under changing environments would directly affect plant growth and biomass accumulation. In the present study, we found that the belowground biomass of *G. uralensis* increased with decreasing belowground N:P, soil C:P, soil N:P and also green leaf C:P, while the belowground biomass of *P. centrasiaticum* improved with reducing soil N:P, green leaf C:P and also green leaf N:P, respectively. Since high C:P and N:P have been considered as more P limited than N^27^, these negative relationships may further prove that increasing P addition alleviates soil P limitation. Thus, biomass accumulation is promoted in belowground organs of both species through its negative influences on soil C:P and N:P. The results broadly suggest that the elemental stoichiometry both in soils and in plants play important roles in driving plant biomass production. However, more field experiments are needed to test whether this statement holds in natural communities.

## Conclusion

Numerous studies have verified that chronic N deposition induces the imbalance of N:P and the increase of P limitations in grasslands^46,47^. Although the monitored N deposition amount in desert steppe is lower than the data reported in other ecosystems^3^, low soil N availability and a critical load of N deposition may result in high sensitivity to chronic and low-level N deposition in a desert steppe. Our results showed that increasing P addition enhanced belowground P of both species and thus altered belowground C:N:P stoichiometry, which was tightly linked to soil and leaf C:N:P stoichiometry. Moderate P addition regulated the balance between soil P supply and plant P demand, consequently, alleviating N-induced P limitations for plant growth. The self-adjusting N strategy of *G. uralensis* might make it keep high inherent stability of C:N:P stoichiometry despite of great stoichiometric change in soils. Our results can provide scientific basis for adaptive management of fragile ecosystems under global climate change. However, the present study was based on a pot-cultured experiment and only performed for three years. Long-term simulated field experiments that test the comparisons among multiple growth forms are urgently needed to further improve our understanding of how plant belowground stoichiometry responds to altering environments and of the adaptation of fragile ecosystems to global climate change.

## Materials and Methods

### Study site

This study was conducted at Sidunzi Grassland Research Station, Yanchi County (37°64′N, 106°50′E, 1450 m a.s.l), Ningxia Hui Autonomous Region, China. This region lies on the southwest edge of the Mu Us Sandy Land and has a typical continental climate in the moderate temperate zone. Mean annual precipitation is 289 mm, with over 70% of it occurring in the growing season (May to September). Mean annual evaporation is 2132 mm. Mean annual temperature is 7.7 °C and mean monthly temperatures range from −8.9 °C in January and 22.5 °C in July. The major soil is classified as Aridisol (FAO classification). The study site has been fenced from domestic animals since 2001. The plant community is chiefly composed of *Lespedeza potanimill*, *P. centrasiaticum*, *G. uralensis*, *Sophora alopecuroides, Cleistogenes squarrosa*, *Agropyron cristatum*, *Stipa capillata*, *Oxytropis aciphylla*, *Caragana intermedia*, *Cynanchum komarovii* and other grasses and shrubs.

### Experimental design

The selected species were *G. uralensis* and *P. centrasiaticum*, both of which are typical perennial species for livestock pasture, wind prevention and sand fixation in northwestern China. *G. uralensis* is an N-fixing species and its roots can build symbiotic relationships with nodule bacteria. *P. centrasiaticum* is a grass and is characterized by functional cross rhizomes. Due to their important economic and ecological value, the plantation of *G. uralensis* and *P. centrasiaticum* have been developed since the early 21^th^ century.

In early April 2011, 36 polyvinyl chloride pots (each 160 mm in diameter and 500 mm in height) were vertically buried into soils with about 50 mm remaining above ground level. Each pot was filled with about 10.0 kg of soils collected from the native habitats of the two studied species. The basic soil properties were measured before transplantation. The organic C, total N, total P, NH_4_^+^-N, NO_3_^−^-N, available P and pH were 1.89 g kg^−1^, 0.22 g kg^−1^, 0.31 g kg^−1^, 1.06 mg kg^−1^, 8.26 mg kg^−1^, 13.16 mg kg^−1^ and 8.53, respectively. *G. uralensis* and *P. centrasiaticum* were planted together in each pot and two seedlings were selected for each species. All seedlings of each species were similar in morphology, specifically 10–12 cm in height and 0.4–0.5 cm in basal diameter and 5–6 in leaf number for *G. uralensis* while 8–10 cm in height and 0.3–0.4 cm in basal diameter and 4–5 in leaf number for *P. centrasiaticum*, respectively. After two weeks, the healthier individual of each species was kept in each pot.

To determine to what extent P addition might alleviate N-induced P limitations for plant growth, six N:P levels were designed and three replicates were chosen for each level. The six levels were treated with the same N amount (10 g N m^−2^ year^−1^) but with different P amounts: 1, 2, 4, 8, 16 and 32 g P m^−2^ year^−1^, thus producing six experimental N:P supply treatments (N10P1, N10P2, N10P4, N10P8, N10P16 and N10P32, respectively). N and P fertilizers were uniformly applied to each pot from May to August during 2011–2013. In order to increase the utility of both fertilizers and avoid high P poison situations, NH_4_NO_3_ and KH_2_PO_4_ were dissolved into water and were added 2–4 times per week. All pots were placed in a flat field and thus received the same precipitation, temperature, light and other environmental factors.

### Field sampling

In late August of 2013, the belowground organs of each species were harvested (roots for *G. uralensis* and rhizomes for *P. centrasiaticum*, respectively). Thirty fully expanded green leaves and the rest of the aboveground organs of each species were also collected. Soils were randomly sampled with an auger at a depth of 0–10 cm. All samples were stored in an insulated can and immediately taken to the laboratory for further analysis.

### Laboratory methods and calculations

Belowground organs of each species were rinsed with distilled water and dried in an oven set to 75 °C for 48 hours to measure belowground biomass per pot. Green leaves and the rest of aboveground organs were dried at 65 °C for 48 hours to measure aboveground biomass per pot. RSR of each species was calculated with the ratio between belowground biomass and aboveground biomass. Total C, N and P concentrations were analyzed after samples were finely ground in a Wiley Mill and passed through a 40-mesh sieve. Total C was measured using the K_2_MnO_4_ volume method, while total N was analyzed with the Kjeldahl acid-digestion method using an Alpkem AutoAnalyzer (Kjektec System 2300 Distilling Unit, Sweden) and total P was determined colorimetrically at 700 nm after reaction with a molybdenum-antimony solution, respectively.

Soil samples were divided into two parts with the first part analyzed for organic C (K_2_MnO_4_ volume method), total N (Kjeldahl acid-digestion method) and total P (HCLO_4_-H_2_SO_4_ method) after air drying. The second part was for the immediate analyses of NH_4_^+^-N and NO_3_^−^-N (both Discontinuous Flow Analyzer, Germany) and available P (NaHCO_3_ method).

### Statistical analysis

SPSS version 13.0 (SPSS Inc., Chicago, IL, USA) was used for statistical analyses. The K-S test was used to confirm normality. The two-way ANOVA was performed to test the effects of N:P supply treatments and species and their interaction on belowground biomass and C:N:P stoichiometry. The one-way ANOVA was performed to test the effects of N:P supply treatments on aboveground biomass, belowground biomass, RSR, belowground C:N:P stoichiometry, green leaf C:N:P stoichiometry and also soil C:N:P stoichiometry, respectively. The independent T test was performed to determine the differences in belowground biomass and C:N:P stoichiometry between the two species under each N:P supply treatment. Linear regression analysis was used to describe the relationships between belowground C:N:P stoichiometry and biomass, between belowground C:N:P stoichiometry and soil C:N:P stoichiometry and also between belowground C:N:P stoichiometry and green leaf C:N:P stoichiometry, respectively. Data are presented as means ± standard error (SE, *n = *3).
